# Entomologic and Serologic Evidence of Zoonotic Transmission of *Babesia microti*, Eastern Switzerland

**DOI:** 10.3201/eid0807.010459

**Published:** 2002-07

**Authors:** Ivo M. Foppa, Peter J. Krause, Andrew Spielman, Heidi Goethert, Lise Gern, Brigit Brand, Sam R. Telford

**Affiliations:** * Harvard School of Public Health, Boston, Massachusetts, USA; † Connecticut Children’s Medical Center and University of Connecticut School of Medicine, Hartford, Connecticut, USA; ‡ Université de Neuchâtel, Institut de Zoologie, Neuchâtel, Switzerland; § Kantonsspital, Blutspendezentrum, Chur, Switzerland

**Keywords:** *Babesia microti*, *Ixodes ricinus*, human babesiosis, Europe

## Abstract

We evaluated human risk for infection with *Babesia microti* at a site in eastern Switzerland where several *B. microti*–infected nymphal *Ixodes ricinus* ticks had been found. DNA from pooled nymphal ticks amplified by polymerase chain reaction was highly homologous to published *B. microti* sequences. More ticks carried babesial infection in the lower portion of the rectangular 0.7-ha grid than in the upper (11% vs. 0.8%). In addition, we measured seroprevalence of immunoglobulin (Ig) G antibodies against *B. microti* antigen in nearby residents. Serum from 1.5% of the 396 human residents of the region reacted to *B. microti* antigen (>1:64), as determined by indirect immunofluorescence assay (IgG). These observations constitute the first report demonstrating *B. microti* in a human-biting vector, associated with evidence of human exposure to this agent in a European site.

A malaria-like syndrome due to *Babesia microti* infection has been recognized in parts of the northeastern United States for more than three decades (1,2). This protozoon pathogen was first isolated more than half a century earlier from a Portuguese vole (3); the pathogen has since been detected in small mammals and ticks throughout Eurasia (3,4).

Despite its broad geographic distribution, *B. microti* has not been implicated as a cause of human illness in Europe. A host-specific, rodent-feeding tick, *Ixodes trianguliceps*, is widely regarded as the main enzootic vector on that continent. *I. ricinus*, the most common human-biting tick of Europe, transmits the Lyme borreliosis spirochete, tick-borne encephalitis virus, the agent of human granulocytic ehrlichiosis, and *B. divergens*, but *I. ricinus* was believed to be infected only occasionally with *B. microti* (5). This vector-pathogen association may account for the absence of human disease due to *B. microti* (3,6). However, subadult *I. ricinus* ticks feed abundantly on the reservoirs of *B. microti*, such as voles and mice, and appear to be competent vectors for *B. microti* (7). In fact, recent studies indicate that Swiss residents may have concurrent infection with the Lyme disease spirochete and *B. microti* (8) and that the human population of certain parts of Germany is exposed to *B. microti* (9).

Human exposure to *B. microti* may occur more often in Europe than has been recognized. Accordingly, we assessed the potential of zoonotic transmission in eastern Switzerland, where other *I. ricinus–*transmitted infections are present. In particular, we determined how frequently *B. microti* parasites infect *I. ricinus* ticks locally, how infection in ticks is spatially distributed in space, and how frequently the sera of nearby residents react to *B. microti* antigen.

## Methods

### Tick Collection and *B. microti* Detection in Ticks

**Figure 1:**
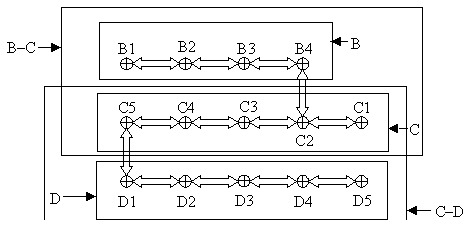
Schematic drawing of the sampling scheme. Distance between sampling points is 30 m. The letters and solid arrows denote the sections used for prevalence estimation. Sampling lines that connect points not belonging to a section are not included in that section (e.g., line B4–C2 is not part of section B).

To assess local prevalence of *B. microti* in host-seeking nymphal *I. ricinus* ticks, we developed a tick-sampling procedure with high spatial resolution (Figure 1). The roughly rectangular, 0.7-ha field site, Ruetiwis (9° 38´ E, 46° 59´ N), is located on a steep southwesterly slope near Seewis in the lower Praettigau Valley of eastern Switzerland at an approximate mean altitude of 850 m above sea level. The site is characterized by abandoned pastures that are partly overgrown by young stands of deciduous and coniferous trees and bushes, as well as by mature mixed forest. All ticks were collected by flagging in July 1997.

To ensure the quality of tick-derived DNA, pools of 2–11 nymphal ticks were transferred to 0.5-mL microcentrifuge tubes containing 50 µL guanidium thiocyanate solution and stored at room temperature until further processing. Before homogenization with a glass pestle, the solution containing the ticks was incubated at 60°C for 2 h. DNA was extracted from the tick homogenate by the phenol-chloroform method. The resulting DNA pellet was suspended in 30 µL DNAse-free H_2_0.

To determine whether *B. microti*–specific DNA was present, the extracted DNA was subjected to polymerase chain reaction (PCR) with the primer pair Bab-1 (5´-ttagtataagcttttatacagc-3´) and Bab-4 (5´-ataggtcagaaacttgaatgataca-3´) (*10*), which targets a 250-bp fragment of the 18s rRNA gene of *B. microti*. After denaturation for 2 min at 94°C, 40 cycles were performed, with 45 sec at 94°C, 45 sec at 55°C, and 45 sec at 72°C, followed by a 7-min final extension. Amplification products were separated on 2% agarose gel in Tris-borate-EDTA buffer, stained with ethidium bromide, and visualized under UV light. To differentiate the sequence of interest from a frequently observed, slightly smaller fragment, purified PCR products were digested with XhoI (Life Technologies, Invitrogen Corp., Gaithersburg, MD). The resulting two fragments specific for *B. microti* migrate in one band on the 2% agarose gel. The corresponding sequence of *B. divergens* lacks the restriction site.

To verify the taxonomic status of the *Babesia* spp. detected in these ticks, we conducted a phylogenetic analysis on a representative sample from which DNA had been amplified (Bab-1/Bab-4). For this purpose we used the primer pair PIRO A and PIRO B, which also targets 18s rDNA but is less specific for *B. microti*; the resulting sequence is longer (400 bp) than the Bab-1/Bab-4 amplicon (11). After PCR amplification, the respective band was excised from the agarose gel, purified with spin columns (Qiagen Inc., Valencia, CA), and sent to the University of Maine sequencing facility for sequence analysis. The resulting sequence was aligned against other *Babesia* spp. listed in GenBank by using Clustal X and consecutive adjustment visually. Phylogenetic analysis was performed by both maximum parsimony (Swofford D. Phylogenetic analysis using parsimony, PAUP*4b61; Sinauer Associates, Inc., Sunderland, MA) and neighbor-joining analyses (12) with *Toxoplasma gondii* (GenBank accession no. X68523) as outgroup. Robustness of the nodes was assessed by bootstrap analysis with 500 bootstrap replicates.

### Data Analysis

To accurately estimate the local prevalence of *B. microti* infection in *I. ricinus* ticks, we developed a maximum likelihood method for point estimation as well as a method for calculating confidence intervals. Briefly, the maximum likelihood estimate (MLE) of the prevalence *p* is based on the likelihood


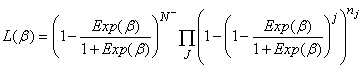
,

where
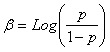
, N^–^ is the number of ticks in negative pools, *J* is the set of pool sizes with at least one positive pool, and *n_j_* is the number of positive pools of size *j*. Test-inversion bootstrap confidence intervals (*13*) were calculated for the prevalence estimate. The method will be described in detail elsewhere (Foppa, unpub. data).

To determine whether infected ticks were clustered in the study site, we tested the null hypothesis *β*_1_ = *β*_2_ = 0 by the model 
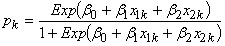
, where *^X^*1*k* = 1 if the pool is from section C and *^X^*1*k* = 0 otherwise, and *^X^*2*k* = 1 if the pool is from section D and *^X^*1*k* = 0 otherwise. If the large sample confidence interval around the MLEs of *β*_1,_
*β*_2_, or both does not include 0, evidence for clustering is positive.

### Serologic Survey

To determine whether humans may be exposed to *B. microti* in the study area, we recruited 400 blood donors living within 10 km of the field site for a serologic survey of tick-borne zoonoses. Volunteers were recruited for this cross-sectional seroprevalence study during blood drives from December 1997 to May 1998 in towns within a 10-km radius of the study site. This protocol was approved by the Human Subjects Committee of the Harvard School of Public Health (protocol number 9712THEE). The sera of participants who gave their written informed consent were tested by indirect immunofluorescence assay (IFA) as described (14). Antigen slides were prepared from erythrocytes of *B. microti*–infected hamsters (the GI strain, originally derived from a Nantucket Island patient). Sera were first screened by IFA at 1:64 dilution. A panel of sera included all samples reactive in the screening test for which enough serum was available, including samples with borderline reactivity and representative controls. This panel was coded and blindly retested for *B. microti* IFA at the University of Connecticut laboratories, which specialize in *Babesia* serology. An IFA titer >1:64 was considered reactive. All reactive sera were titrated to endpoint.

## Results

### Tick Survey

**Figure 2:**
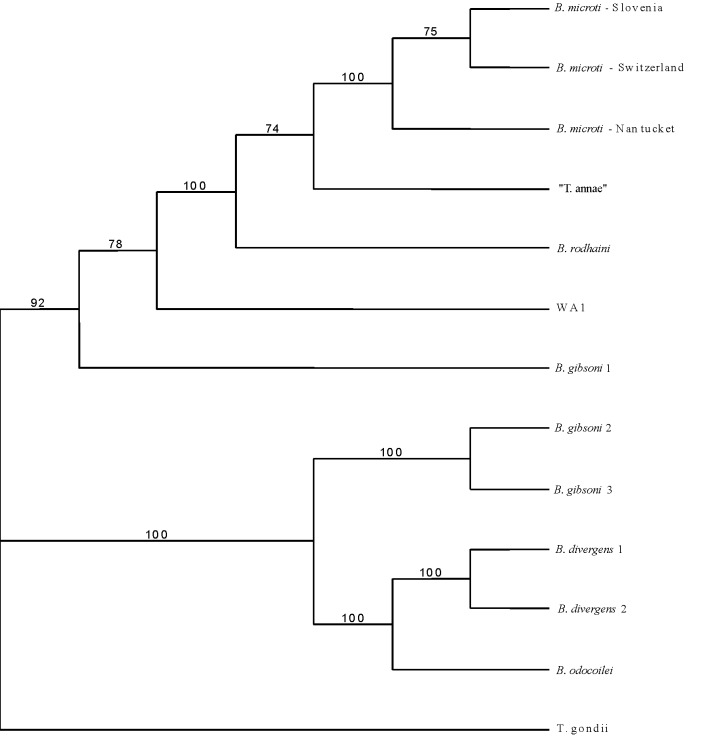
Maximum parsimony bootstrap consensus tree of 18S rDNA. GenBank accession nos.: *Babesia microti*-Slovenia AF373332; *B. microti*-Switzerland AF494286; *B. microti-* Nantucket AF231348; "Toxoplasma annae" AF188001; *B. rodhaini* AB049999; WA1 AF158700; *B. gibsoni* 1 AF158702; *B. divergens* 1 U07885; *B. divergens* 2 U16370; *B. odocoilei* U16369; *B. gibsoni* 2 AF175300; *B. gibsoni* 3 AF175301; and *Toxoplasma gondii* X68523.

To verify the identity of the amplified DNA, a phylogenetic analysis was performed. The sequence amplified from our *I. ricinus* ticks, which was deposited in GenBank (accession no. AF494286), differed by l bp from the North American *B. microti* sequence (GenBank accession no. AF231348) and was identical with that of *B. microti* from Slovenia (GenBank accession no. AF373332). Accordingly, our sequence clearly clustered with the European and the American strain of *B. microti* (Figure 2), with concordant results from both maximum parsimony and neighbor-joining analyses. Therefore, the piroplasms detected in *I. ricinus* ticks from Switzerland must be considered *B. microti.*

We then determined the prevalence of *B. microti* infection in nymphal *I. ricinus* ticks from the study site, on the basis of PCR amplification of *B. microti* in DNA tick pools. Overall, we analyzed 408 ticks in 64 pools. We detected *B. microti–*specific DNA in 14 pools (Table 1). Thus, *B. microti* infection appears to be common in human-biting ticks at this central European study site.

**Table 1:** *Babesia microti* infection in nymphal *Ixodes ricinus* ticks as determined by polymerase chain reaction, eastern Switzerland

Sampling point or line	Pool 1	Pool 2	Pool 3	Pool 4	Pool 5	No. of pools	No. of ticks
B1	11	11	12	—	—	3	34
B1–2	10	10	10	—	—	3	30
B2	4	3	—	—	—	2	7
B2–3	3	3	3	—	—	3	9
B3	3	—	—	—	—	1	3
B3–4	8^a^	8	—	—	—	2	16
B4	1	—	—	—	—	1	1
B4–C2	5	5	4	5	—	4	19
C1	1	—	—	—	—	1	1
C1–2	9	9	9	9	—	4	36
C2	7	7	5	—	—	3	19
C2–3	5	5	5	5	5	5	25
C3	10	—	—	—	—	1	10
C3–4	4	—	—	—	—	1	4
C4	4	—	—	—	—	1	4
C4–5	7	6	—	—	—	2	13
C5	8	7	6	—	—	3	21
C5–D1	8	7	7	8	—	4	30
D1	5	5	6	—	—	3	16
D1–2	8	7	—	—	—	2	15
D2	6	6	—	—	—	2	12
D2–3	5	5	5	6	6	5	27
D3	4	—	—	—	—	1	4
D3–4	6	6	5	—	—	3	17
D4	11	11	10	—	—	3	32
D4–5	3	—	—	—	—	1	3
D5	0	—	—	—	—	0	0
Total						64	408

^a^Pools in bold are positive; dashes represent the absence of further pools.

To determine whether the prevalence of *B. microti* is distributed homogeneously within the study site, prevalence of infection in ticks was estimated for selected segments of the sampling grid. The overall prevalence of *B. microti* infection in ticks, as estimated by MLE, was close to 4%. More detailed spatial analysis, however, indicates that the distribution of babesial infection at the site is heterogeneous (Table 2). In the lower portion of the site (section D), >10% of all ticks were infected, while in the upper portion (B–C), prevalence was <1%. Prevalence of infection in ticks was similar in sections B and C (p=0.161), but greater in section D than in sections B and C (p=0.003). Thus, *B. microti* transmission is focal at this site.

**Table 2:** Global and local prevalence estimates of *Babesia microti* infection in nymphal *Ixodes ricinus* ticks as determined by polymerase chain reaction, eastern Switzerland

	Point estimate (%)	95% confidence interval
MLE^a^, overall	3.6	0.2 to 9.06
MLE, section B	1.04	0.10 to 9.01
MLE, section C	0.77	0.08 to 8.47
MLE, section D	10.99	6.17 to 17.71
MLE, sections B,C	0.82	0.61 to 2.65
MLE, sections C,D	5.25	2.96 to 10.43

^a^MLE (maximum likelihood estimate) of the point estimates are shown with 95% bootstrap confidence limits.

### Serologic Survey

To determine how frequently humans are exposed to *B. microti* infection in the region around the study site, sera of local residents were screened by IFA for the presence of antibodies to *B. microti* antigen. Most sera (80%) were collected from December through March, when *I. ricinus* is not active. Five (1.5%) of 396 human sera were immunoglobulin (Ig) G-reactive against *B. microti* antigen at a titer ³1:64. All the samples positive on initial testing and one with a borderline result were confirmed on retesting to be reactive at a titer >1:64, while none of the samples with a negative screening result reacted (Table 3). *B. microti–*specific IgM seroreactivity was not found in any of the sera, a finding compatible with the time of the year when most of the sera had been collected. These results indicate that residents of our central European study site are exposed to bites of ticks infected with *B. microti*.

**Table 3:** Reactivity of sera tested against *Babesia microti* as determined by indirect immunofluorescent assay

Titer	No. of sera (%)
<1:64	391 (98.7)
1:64	1 (0.3)
1:128	1 (0.3)
1:256	1 (0.3)
1:512	2 (0.5)
Total	396 (100.0)

## Discussion

*B. microti–*infected nymphal *I. ricinus* ticks are present at Swiss study site, and human residents of the area appear to be exposed to this agent. However, the presence of *B. microti* in *I. ricinus* ticks has been reported only once before (5).

A phylogenetic analysis clearly demonstrated that the piroplasms found in our study site belong to *B. microti*, rather than to other *Babesia* or *Theileria* species (Figure 2). The detection of *B. microti* DNA in host-seeking nymphal *I. ricinus* ticks may, however, simply reflect “spill-over” from enzootic transmission by the accepted maintenance vector for *B. microti* in Eurasia, the tick *I. trianguliceps* (15). This tick, which does not bite humans, infests small rodents that also are abundantly parasitized by *I. ricinus* (3,16). The prevalence of infection in ticks may be underestimated as only one genome copy is present per parasite in unfed ticks before sporogony. Therefore, low parasite loads may escape detection, thus increasing the specificity of the assay.

In our study, maximizing specificity was desirable because infection of *I. ricinus* with *B. microti* was a priori assumed to be rare, and underestimation of prevalence therefore is conservative. Regardless, the proportion of ticks that appear to be infected by *B. microti* is similar to that in coastal New England (S. Telford, unpub. data). Recently, Duh et al. reported a similarly high prevalence of *B. microti* infection in nymphal *I. ricinus* ticks collected in Slovenia (7 of 69 ticks tested by PCR) (17). In combination with our findings, this report suggests that *B. microti* infection in *I. ricinus* ticks is far more common than traditionally thought. In addition, vector competence of *I. ricinus* for *B. microti* has been demonstrated experimentally (7,18). The frequent infection of *I. ricinus* with this piroplasm therefore implies zoonotic relevance of this vector-pathogen association in Switzerland and possibly in other parts of Europe.

*B. microti* transmission is clustered in the study site. Similar to tick-borne encephalitis virus, *B. microti* seems to be maintained in small focal areas. The risk of human infection therefore is spatially highly variable and may be conditional on tick density. Preliminary analysis of tick infection data from the study site over a 3-year period (Foppa, unpub. data) suggests that *B. microti* is locally maintained, especially in the lower portion of that site, while the exact location of the maximum risk changes over the years.

Residents of this site in eastern Switzerland appear to be exposed to bites by ticks infected with *B. microti*. The serologic result is unlikely to reflect low specificity of the assay, as previous evaluation of this IFA has demonstrated high specificity (19,20). As part of this evaluation, we tested 50 sera from residents of Iceland, where ticks capable of transmitting *B. microti* are absent; none of the sera reacted with *B. microti* antigen (19). We recently repeated testing of these sera and obtained similar results. This finding suggests a satisfactory positive predictive value of the serologic test even in settings of low prevalence.

In the northeastern United States, *B. microti* seroprevalence has varied in endemic regions, from 3.7% in Red Cross blood donors on Cape Cod, Massachusetts (21), to 2.5% and 9.5% in Connecticut residents who were seronegative and seroreactive, respectively, to *Borrelia burgdorferi* (22). Our findings of high local prevalence of *B. microti* infection in *I. ricinus* ticks may seem counterintuitive given the lower seroprevalence in residents of our study area. The findings may, however, reflect a high degree of spatial clustering of transmission with a low average risk (18). Alternatively, the low seroprevalence may be the result of low test sensitivity resulting from antigenic differences between North American *B. microti* strains, which were used for the serologic testing, and the European strains to which our study population had been exposed.

At least locally, the potential for zoonotic transmission of *B. microti* by *I. ricinus* is considerable, which explains serologic evidence in human beings of exposure to this agent in parts of Europe. The lack of recognized human pathology associated with European strains of *B. microti*, despite exposure to infectious tick bites, may be a consequence of a lower virulence of European strains than those of North American. Disease episodes due to *B. microti*, on the other hand, may be overlooked because of the relative nonspecificity of signs and symptoms and the presumption that this agent rarely infects *I. ricinus*.
